# Organizational Entrepreneurship and Administrators of Hospitals: Case Study of Iran

**DOI:** 10.5539/gjhs.v6n3p249

**Published:** 2014-04-11

**Authors:** Mehdi Raadabadi, Ahmad Fayaz-Bakhsh, Aslan Nazari, Seyed Masood Mousavi, MohammadAli Fayaz-Bakhsh

**Affiliations:** 1 Research Center for Health Services Management, Institute for Futures Studies in Health, Kerman University of Medical Sciences, Kerman, Iran; 2 School of Allied Medical Sciences, Tehran University of Medical Sciences, Tehran, Iran; 3 Health Management and Economics Research Center, Iran University of Medical Sciences, Tehran, Iran; 4 Hospital Management Research Center, Iran University of Medical Sciences, Tehran, Iran; 5 Islamic Azad University-South Tehran Branch, Tehran, Iran

**Keywords:** entrepreneurship, hospitals, creativity, health system, healthcare activities, hospitals’, manager

## Abstract

Due to rapid changes of technology and scientific advances in health systems and need for fast planning in health care, entrepreneurial spirit among employers and employees is a crucial element. According to the field of entrepreneurship research has not been solved and where learning and innovation for healthcare organizations due to the nature of the work required.

This study aims to examine the entrepreneurial activities within the hospitals affiliated to Tehran University of Medical Sciences, Iran. To achieve the aim of the study, a questionnaire containing 29 items regarding the areas of innovation, creative behavior, flexibility, empowerment, rewarding systems and the management support was distributed among the hospitals’ managers. Establishment of a culture of entrepreneurship in healthcare organizations led to the development unit controlled, changing the culture of the hospital.

The analysis of the data showed that the majority of the managers agreed with all five areas of entrepreneurship namely the existence of innovation and innovative behavior, flexibility, decision making, rewarding and encouraging system, as well as management supportive system of personnel’s new ideas. In fact, the managers generally had positive attitude towards entrepreneurship in their organizations The Pearson correlation test also showed that there is a significant relationship between the areas of entrepreneurship and the managers’ age as well as their working experience (P<0.05).

Entrepreneurial activities in healthcare can be improved through providing a suitable environment, adjusting reward and encouragement systems, giving more authority to subordinates, promoting awareness and education, and mobilizing managers to attract appropriate opportunities for organization. Further active involvement of employees, more stable in front of changes and increased ability managers to capture opportunities in domestic and foreign situation.

## 1. Introduction

Today, organizational environment is so complex and uncertain that institutions can no longer survive through superficial changes such as changes in the method, system and structure (Amirkabiri & Fathi, 2010). In the recent years, several attempts have been made in the field of competition with the aim of detecting entrepreneurs who have capabilities in creating new workflows, in creative problem solving and in developing other necessary capacities (Ernesto-Amoros et al., 2010; Alimardani & Ghahramani, 2009).

Entrepreneurship is a significant source of social mobility that covers various subjects, but the problem is that many areas of entrepreneurship in research are still virgin (Luke & Verreynne, 2006). Some studies have shown that about one third of America’s workforces in business are women showing that gender does not play a significant role in creating entrepreneurship (Peris-Ortiz et al.,2012). Entrepreneurship is one of the most efficient methods of operation and management of change, which the motion of the operation (bureaucracy) is converted into the attitude, culture and entrepreneurial management (Jahangiri & Kalantari-Saghafi, 2008).

Learning and innovation are fundamental requirements for organizations that are seeking effectiveness and survival. Many organizations are also looking for highly innovative and entrepreneurial approaches to improve their effectiveness, efficiency and flexibility (Haghshenas et al.,2008). Based upon previous conducted research studies and evidences about management and policy in Latin American countries, to achieve economic and entrepreneurship dynamism, and to become innovative companies with high added value, the policy makers need to establish entrepreneurial culture (Ernesto-Amoros et al.,2012). The importance and capabilities of organizational entrepreneurship is fully perceptible because employees give new value to the services and products offered by the organization, and this highlights the learning, creation and innovation synchronization that will not be complete without any information factor (Vilaseca-Requena et al., 2007). Entrepreneurship is like an environmental organization in which creativity and innovation of staff are blossomed, and issues like communications and information technology flourish more by providing timely and uniform distribution of the information (Antoncic, 2007). Every year, entrepreneur world scout examines the rates of entrepreneurial activities and barriers to entrepreneurship by implementing field research in member states. These cases represent a global effort on this issue on the basis of offering the practical recommendations and encouraging the exchange of best practices and benchmarking so that it provides a basis for synergy and strengthens entrepreneurship development programs at the country level (Bosma et al.,2008). In general, the processes of organizational entrepreneurship in private and public organizations can be presented for nonprofit organizations. Researchers in various disciplines have studied public entrepreneur-ship, but there is little work in management and economics on the natur (Klein, Mahoney, McGahan, & Pitelis, 2010).

Due to rapid changes in technology and scientific advances among health care provider organizations, determining the future needs and planning them appropriately are very difficult. Health care systems like other economic organizations in the world are very complex and chaotic in which traditional approaches no longer apply. Lack of accurate planning and management in these systems will limit the expectations and creativity which is required to solve complex and new prospective problems of health care. Accordingly, for the conservation and sustainability of the organizations in the era of development and reconstruction of health care systems, innovation and entrepreneurship are a prerequisite (Asefzade & Rezapor, 2007).

Factors such as increasing costs of services, competition, expensive equipment, old population and high cultural diversity affect health services environment. Healthcare related organizations exposed to these challenges are more complex; therefore, they seek solutions for their long-term survival. Achieving such solutions wont be possible without change, innovation and an entrepreneur attitude (Robey, 1998). Different experts have offered numerous scientific and practical frameworks for the study of entrepreneurship within the organization. Many researchers have examined the results of entrepreneurship within organizations and its different dimensions. The growth and profitability of the organizations as well as the customers’ satisfaction have been the principal dimensions of entrepreneurship outcomes (Antoncic & Hisrich, 2001). According to the relevant previous studies conducted in this field and due to the importance of managers’ viewpoints and their performances in promoting the entrepreneurial culture, learning and innovation for healthcare organizations with regard to the nature of their activities are an essential need. In addition, rapid technological changes and scientific advances in healthcare systems and the need for immediate planning in healthcare will necessitate the presence of entrepreneur managers in this area. The study aims to examine the business activities in hospitals but it seems there is an analysis of point of view of managers about the possibility/need to develop a more organizational entrepreneurship

## 2. Method

This cross-sectional study was conducted among senior and middle managers in 10 selected hospitals affiliated to Tehran University of Medical Sciences (TUMS), Iran. Study population was all senior and middle executives, including chairmen and chief executives, directors of hospital financial departments, directors of administrative-support departments, senior supervisors, as well as training and clinical supervisors in the hospitals affiliated to TUMS. Ten hospitals were randomly selected from among all 26 hospitals affiliated to TUMS. Data collection tool was a reliable and validated questionnaire consisting of two main parts. The content validity and face validity of the questionnaire were checked by two experts in the field based upon two designed forms (content validation form and face validation form). Moreover, to see the internal reliability of the questionnaire, Cronbach’s alpha coefficient was calculated. The coefficient was found to be 0.77 (Yadolahi-Farsi et al.,2008). The obtained coefficient indicated a good internal reliability for the the questionnaire.

The first part of the questionnaire deals with the respondents’ demographic information including age, sex, years of working experience and education level. The second part included 29 items in the areas of innovation, creativity, flexibility, empowerment, reward systems and the management support. The items were designed on a five-point Likert scale of agreement, where 1 = strongly disagree, 2 = disagree, 3 = undecided, 4 = agree, and 5 = strongly agree. While distributing the questionnaires, more explanations were given to the respondents regarding the aim and scope of the study, as well as how the questionnaire should be filled out. Furthermore, if the respondents were interested in the research results, they were assured that the findings will be sent to them after analyzing the obtained information. Moreover, the respondents were given enough time to fill out the questionnaire. At the end, the collected data was entered into the SPSS software, version 18, and then data analysis was performed using the t-test, two-way ANOVA and Pearson correlation test.

## 3. Results

This study was conducted in 10 hospitals affiliated to Tehran University of Medical Sciences. In total, 120 questionnaires were distributed among the managers but 95 questionnaires were returned. About 70 % of the respondents were female and the rest of 30 % surveyed managers were male. The age distribution showed that about half of the surveyed managers were in the age range of 40-49 (45.6%). Most of the respondents had bachelor’s degree and they had managerial experience between 10 to 20 years (see Table 1).

**Table 1:** Frequency distribution of managers in terms of demographic characteristics

Variable		Number	Percent
Age range	20-29	8	8.9%
	30-39	32	32.8%
	40-49	44	45.6%
	50-59	11	12.7%
Educational degree	Diploma	2	2.2%
	Associate`s degree	2	2.2%
	BA	67	68.8%
	MA	17	18.2%
	Ph.D.	7	8.6%
Work experience in managerial posts	0-5	13	13.4%
	6-10	10	10.4%
	11-15	28	28.4%
	16-20	28	29.9%
	21-25	16	17.9%

The results showed that the majority of the managers agreed with all five areas of entrepreneurship namely the existence of innovation and innovative behavior, flexibility, decision making, rewarding and encouraging system, as well as management supportive system of personnel’s new ideas. In fact, the managers generally had positive attitude towards entrepreneurship in their organizations (see Table 2).

**Table 2:** Average areas related to entrepreneurship among the managers of the hospitals

Areas	Strongly agree & Agree	Undecided	Disagree & Strongly disagree
Level of innovation and innovative behavior	46.5 %	32.3%	21. 2 %
Level of flexibility	55.6 %	23.8%	20.6 %
Decision making	63 %	21.4%	15.6 %
Rewarding and encouraging system	45 %	32.8%	22.2 %
Management supportive system of personnel’s new ideas	45.4 %	33.6 %	21. %
Average	51.1 %	28.8%	20.1 %

The t-test did not show any significant relationships between the managers’ gender and the areas under investigation. The Pearson test showed that, except for the second area namely level of flexibility, there is a significant relationship between managers’ working experience and the areas under investigation (see Table 3).

**Table 3:** Relationship between the areas of entrepreneurship and managers’ working experience

Areas	Pearson	P value
Level of innovation and innovative behavior	-0.359	0.003
Level of flexibility	-0.22	0.072
Decision making	-0.25	0.040
Rewarding and encouraging system	-0.32	0.008
Management supportive system of personnel’s new ideas	-0.37	0.002

The Pearson negative value indicates a negative relationship between the areas under investigation and the managers’ working experience. In other words, younger managers agree much more with entrepreneurship within their organizations.

Table 4 shows the relationship between the managers’ age and the areas of entrepreneurship. Pearson test showed that there is a negative. In other words, the level of entrepreneurship is lower in the older managers. In total, the overall average of the five areas under investigation showed that most of the managers agree with entrepreneurship in their hospitals.

**Table 4:** Relationship between the areas of entrepreneurship and managers’ age

Areas	Pearson	P value
Level of innovation and innovative behavior	-0.36	0.001
Level of flexibility	-0.27	0.016
Decision making	-0.26	0.021
Rewarding and encouraging system	-0.23	0.040
Management supportive system of personnel’s new ideas	-0.30	0.008

## 4. Discussion

### 4.1 Level of Innovation and Innovative Behavior

In this area, less than half of the respondents (46.52%) agreed with the innovation and innovative practices in their organizations. However, about one-fifth of the surveyed managers (21.12%) disagreed with this level of entrepreneurship in their organizations. Therefore, this factor can be regarded as an important factor in entrepreneurship in the hospitals. The results of this study are in accordance with the findings of Reisi et al. (2008) in which they found that creativity and innovation are effective factors in entrepreneurship. Moreover, Tafazoli (1994) stated that the major reason for economic backwardness in developing countries is the lack of individual creativity understanding. He pointed out that although the human spirit for success is formed in childhood, it is possible to make a creative spirit and business mission in people by proper training programs. Without any doubt, both innovation and entrepreneurship are essential components for obtaining desirable results.

### 4.2 Level of Flexibility

In this area, more than half of the surveyed managers (55.60 %) agreed with the flexibility in their organizations. Sean (2005) also referred to a direct relationship between flexibility and improvement of organizations’ performance and considered it as an effective factor in innovation and doing innovative practices. The concept of flexibility in the last decade has attracted a great deal of researchers’ attentions. This concept has been defined as the organization’s ability to respond to the diverse demands of its dynamic competitive environment (Ngo & Loi, 2008). Although the concept of flexibility covers both the employees and the structure of organizations, this concept is a subset of entrepreneurship. Accordingly, the success of an organization in entrepreneurship directly depends upon the level of flexibility in that organization.

### 4.3 Decision Making

In the area of considering empowerment as an encouragement to the employees, about two-third (63%) of the respondents agreed to give authority to managers and lower level employees. In his study, Shirzad (2001) concluded that implementation of empowerment programs attract managers’ attention to the basic objectives, accelerate decision-making and increase motivation. Moreover, Shahab (1999) stated that empowerment is the second priority which management needs for doing better administrative and executive activities. Therefore, it can be concluded that entrepreneurship in organizations with empowerment raises the participation rate among the employees and increases their motivation to perform better so that it can be considered as one of the important pillars of entrepreneurship within the organization

### 4.4 Reward and Encouragement System

With regard to the hospitals equipped with rewarding and encouraging systems for the entrepreneurial ideas, less than half of the surveyed managers (45%) welcomed the presence of this system. However, 22.13% of the respondents disagreed with this system in the hospitals. In general, rewarding system in entrepreneurial organizations has its own characteristics. Practicality and feasibility of the considered reward is one of them. For example, bonuses considered for the employees should be payable. The rewards should also be available for the employees. In this regard, various incentives should be employed. Both financial and non-financial incentives are important, but more intrinsic encouragements should be used (Cornwall & Perlman, 1990). Sykes and Block (1989) believe that organizations must consider a rewarding system to overcome the bureaucratic barriers and move towards entrepreneurship with more emphasis on giving the reward on the basis of employees’ performances. Providing appropriate encouragement and rewarding systems is one of the most important factors in entrepreneurial organizations. Traditional methods of promotion and reward can rarely be effective for entrepreneurs (Stevenson & Gumpert, 1985; Thompson, 1999). Using appropriate rewards can raise employees’ desire to accept the risks associated with entrepreneurial activities. Thus, although reward system is supposed to be simple, several studies have shown that there is a direct relationship between the level of entrepreneurial organizations and reward system. In this study the majority of administrators agreed with this system in their organizations.

### 4.5 Management Supportive System of Personnel’s New Ideas

In this area, less than half of the surveyed managers (45.39 %) believed in the presence of management supportive system of personnel’s new ideas in their organizations. According to Paolini (1991), supporting the personnel is considered as one of the five major steps of entrepreneurship development process within organizations. In his study, Yusuf (1995) concluded that one of the critical factors affecting success of entrepreneurship is the organizational support such as providing infrastructures and training facilities. This study indicated that there is a significant relationship between managers’ age and each areas of entrepreneurship. In this regard, Knight (1997) determined a set of criteria for measuring entrepreneurial tendencies that show a negative relationship between the managers’ age and taking risks. Moreover, in his study, Ahmadpor-Daryani (2000) showed that younger people need to succeed more than older people. With regard to the managers’ working experience, except for the second area namely level of flexibility, there was a significant relationship between managers’ working experience and the entrepreneurial areas under investigation.

In their study, Hornsby et al. (2002) concluded that there is a negative relationship between managerial experiences and the amount of managers’ entrepreneurial activities. They stated that more experienced managers are more cautious than younger managers in dealing with organizational issues. Moreover, in their study on middle level managers, Floyd and Woolridge (1994) found that there is a negative relationship between managerial experiences of middle level managers and their tendency to do entrepreneurial activities; however, this relationship was not highly significant. All in all, the results of this study and the findings of other related studies illustrate the importance of the five areas of study in entrepreneurship. Furthermore, it was shown that each of these factors is necessary to implement entrepreneurship in an organization such as a hospital.

## 5. Conclusion

It can be concluded that the more hospitals’ managers strengthen the characteristics associated with entrepreneurship by giving value to their entrepreneurial ideas, the better they can develop entrepreneurial activities within their respective hospitals. This also leads to more staff participation within the hospitals. The process of entrepreneurial activities can be improved by changing the culture of hospitals through providing a suitable environment for creative people, creating a system of reward and encouragement, giving more authority to subordinates, promoting personnel’s awareness, training the personnel in the field of working values and responsibility, and increasing managers’ ability to absorb appropriate opportunities for organization. In the areas such as healthcare services, organizations can be more irresponsive against changes, because the entrepreneurial organization is an organization that is capable to strengthen creativity and innovation according to environmental changes and structural complexity. In organizational entrepreneurship, the structural dimensions are regarded as the important issues that seriously prevent the spread of entrepreneurship and act as a barrier against the employees. Several studies have shown that there is a negative relationship between entrepreneurship and complexity in the organization because complexity reduces the relationship among the group members within the organization. Moreover, there is a negative relationship between entrepreneurship and formality because the personnel’s formality reduces the flexibility and freedom within an organization. For creating entrepreneurial activities within the organizations such as the hospitals, it is required to remove the barriers, such as structural complex aspects. Bureaucracy in organizations must be at the lowest level; however, due to the existence of bureaucracy in most of the Iranian organizations, it may be a little hard to imagine basic change in their structures. Nevertheless, in order to keep pace with the changes, particularly the global changes, considering the above mentioned entrepreneurial activities and their interactions seems inevitable.
